# The clinical value of ultrasound parameters combined with clinical indicators in predicting embryo implantation rates in polycystic ovary syndrome patients at different body mass index levels

**DOI:** 10.3389/fendo.2026.1824366

**Published:** 2026-08-11

**Authors:** Chenhuizi Wu, Hanyue Zhang, Liuyun Cai, Yanxu Lu, Huijia Chen, Xiaodong Luo

**Affiliations:** Department of Gynecology and Obstetrics, The Second Affiliated Hospital of Chongqing Medical University, Chongqing, China

**Keywords:** body mass index, embryo implantation rate, endometrial receptivity, *in vitro* fertilization-embryo transfer, obese, polycystic ovary syndrome

## Abstract

**Objective:**

To evaluate the predictive value of endometrial and uterine artery blood flow parameters, combined with clinical indicators, for embryo implantation outcomes in PCOS patients undergoing IVF-ET across different BMI categories.

**Methods:**

We retrospectively analyzed 129 PCOS patients who underwent IVF-ET at our hospital from June 2022 to June 2025. Participants were stratified into four BMI groups. Transvaginal ultrasound parameters and clinical data were statistically analyzed.

**Results:**

Significant differences were observed in endometrial thickness, morphological patterns, blood flow distribution, and uterine artery blood flow parameters across different BMI groups. The obese group exhibited a tendency toward thinner endometrium, a lower proportion of type A endometrial morphology, reduced endometrial perfusion, and a higher uterine artery resistance index. Logistic regression analysis identified high endometrial blood flow grade, abundant endometrial blood flow, and a greater number of high-quality embryos as protective factors for successful embryo implantation following IVF-ET in patients with PCOS (all P<0.05). Conversely, advanced age was associated with an increased risk of implantation failure (P<0.05). ROC curve analysis demonstrated that a multivariable model incorporating endometrial blood flow grade, endometrial blood flow quantity, number of high-quality embryos, and age had strong predictive performance for implantation success (AUC = 0.917).

**Conclusion:**

Weight management before assisted reproduction is clinically important. Overweight patients should monitor endometrial and uterine artery blood flow parameters and receive targeted interventions to improve pregnancy outcomes.

## 1 Introduction

Polycystic ovary syndrome (PCOS), which affects approximately 8%–13% of women of reproductive age, is a leading cause of anovulatory infertility (1). Notably, 38% to 88% of patients with PCOS are overweight or obese, and elevated body mass index (BMI) is known to adversely affect reproductive function and pregnancy outcomes. In the context of assisted reproductive technology, endometrial morphological features and hemodynamic parameters serve as crucial indicators of endometrial receptivity during *in vitro* fertilization and embryo transfer (IVF-ET) cycles, with strong clinical relevance to pregnancy success. Research indicates that obesity is a significant contributor to impaired endometrial receptivity and diminished oocyte quality (2). This study focuses on PCOS patients across different BMI categories. We systematically evaluate the impact of BMI on endometrial receptivity and uterine artery blood flow during IVF-ET treatment cycles and assess the ability of combined ultrasound and clinical indicators to predict embryo implantation rates. The findings aim to offer evidence-based insights for optimizing individualized treatment protocols and improving clinical pregnancy outcomes.

## 2 Materials and methods

### 2.1 Study population and design

**Figure 1:**
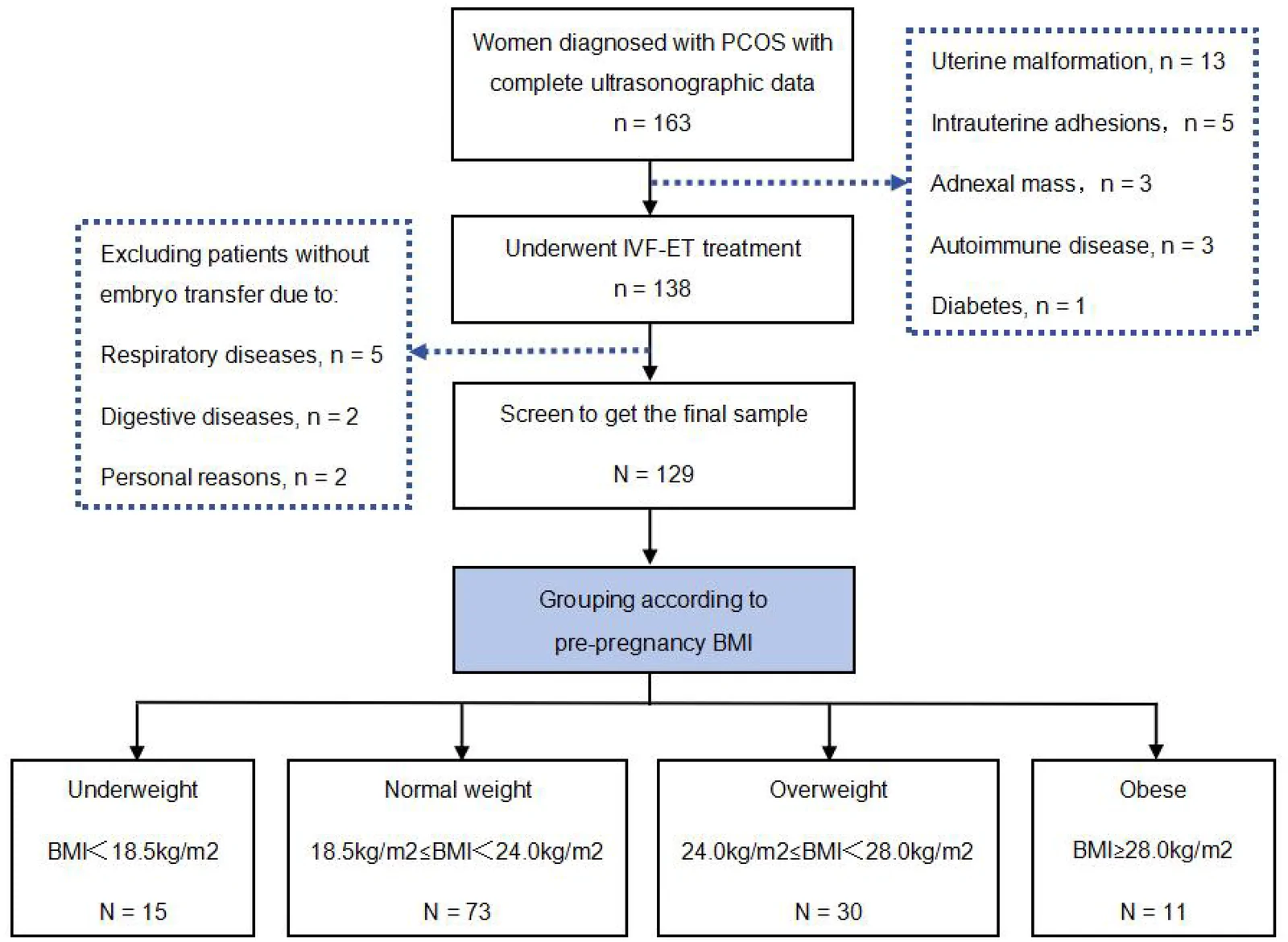
Study flow chart.

This retrospective study enrolled 129 patients with PCOS who underwent *in vitro* fertilization and embryo transfer (IVF-ET) treatment at the Reproductive Medicine Center of the Second Affiliated Hospital of Chongqing Medical University between June 2022 and June 2025. The patient selection process is illustrated in **Figure 1**. Participants were categorized into four groups according to body mass index (BMI) based on established criteria (3): underweight (<18.5 kg/m², Group A), normal weight (18.5–24.0 kg/m², Group B), overweight (24.0–28.0 kg/m², Group C), and obese (≥28.0 kg/m², Group D).

Inclusion criteria were as follows: (1) diagnosis of PCOS according to standard criteria (4); (2) diagnosis of infertility as defined clinically (5); (3) undergoing IVF-ET with frozen-thawed embryo transfer; and (4) availability of complete transvaginal ultrasound data and pregnancy outcome records.

Exclusion criteria included: (1) concurrent uterine abnormalities such as adenomyosis, congenital uterine malformations, or intrauterine adhesions; (2) coexisting ovarian pathologies including ovarian cysts or tumors; (3) tubal factor infertility or abnormal semen analysis of the partner; (4) systemic diseases such as diabetes, hematologic disorders, autoimmune conditions, acute or chronic infections, or severe hepatic or renal dysfunction; (5) chromosomal abnormalities in either partner; and (6) patients who received any investigational or adjunctive treatments to improve endometrial receptivity, such as Platelet-Rich Plasma (PRP) therapy or granulocyte colony-stimulating factor (G-CSF) infusion, prior to embryo transfer. The study protocol was approved by the Ethics Committee of our hospital (Approval No. 2023686). Human ethics and consent to participate declarations:not applicable.

### 2.2 Research methods

#### 2.2.1 Ultrasound procedures

Ultrasound examinations were conducted using a Color Doppler ultrasound system (GE Voluson E8/E10) equipped with a transvaginal probe (RIC5-9-D, frequency range 5–9 MHz). Patients were positioned in lithotomy, and the transvaginal probe was gently inserted after the bladder was emptied. Endometrial morphology was initially classified according to the Gonen criteria (6): Type A: distinct trilaminar pattern with hyperechoic outer lines and a hypoechoic central cavity; Type B: intermediate echogenicity with partial loss of the trilaminar appearance; Type C: homogeneous hyperechogenicity without visible layering. Endometrial thickness was subsequently measured. The sagittal uterine view was then examined to identify the hypoechoic zone at the endometrial–myometrial junction and the region of maximal blood flow signal on Color Doppler imaging. The number of vessels penetrating the endometrium and the endometrial blood flow grade (assessed using the Applebaum grading system (7)) were documented. The uterine artery Doppler spectrum was recorded, and the built-in automated analysis software was used to calculate the systolic-to-diastolic velocity ratio (S/D), resistance index (RI), and pulsatility index (PI). Each parameter was measured in triplicate, and the mean value was used for final analysis. All scans were performed by two experienced sonographers, each with over five years of clinical practice.

#### 2.2.2 IVF-ET protocol

##### 2.2.2.1 Oocyte retrieval, fertilization, and embryo transfer

All patients underwent controlled ovarian stimulation with an individualized protocol. The regimen (GnRH antagonist, long luteal-phase, or mild stimulation protocol, among others) was selected based on the patient’s clinical profile. Follicular development was monitored by serial transvaginal ultrasound. Final oocyte maturation was triggered with a single intramuscular injection of 10,000 IU human chorionic gonadotropin (hCG) when at least one of the following follicular size criteria was met: (1) one follicle ≥18 mm, (2) two follicles ≥17 mm, or (3) three follicles ≥16 mm in diameter. Transvaginal ultrasound-guided oocyte retrieval was performed 36 hours after hCG administration. Retrieved oocytes were immediately fertilized via conventional *in vitro* fertilization (IVF). All resulting embryos were cryopreserved (full embryo cryopreservation).

##### 2.2.2.2 Embryo grading

After 24 hours of *in vitro* culture, all zygotes were assessed morphologically. Embryos were graded according to the following criteria: Grade I (High quality): Blastomeres of equal size and regular shape; clear, homogeneous cytoplasm; intact zona pellucida; fragmentation <5%; 6–10 cells. Grade II (High quality): Slightly uneven blastomere size; mildly irregular shape; fragmentation 5%–20%; 6–10 cells. Grade III: Uneven blastomere size; irregular shape; coarse granular cytoplasm; fragmentation 21%–49%. Grade IV: Highly uneven blastomere size; multinucleation present; prominent cytoplasmic vacuolization; fragmentation >50%. Embryos graded I or II were defined as high-quality embryos. On the afternoon of day 3 post-retrieval, suitable cleavage-stage embryos were placed in blastocyst culture medium. Blastocyst development was assessed on days 5 and 6. According to the Gardner blastocyst grading system (8), blastocysts scoring 3BB or higher were selected for cryopreservation.

##### 2.2.2.3 Endometrial preparation protocols

###### 2.2.2.3.1 Stimulated cycle protocol

Starting on menstrual cycle day 4–5, patients received oral letrozole 2.5 mg once daily. From day 10 onward, urinary gonadotropin (75–150 IU) was administered intramuscularly once daily, with dosing adjusted based on follicular response as monitored by transvaginal ultrasound. Monitoring continued until a dominant follicle reached ≥18 mm in diameter and endometrial thickness was ≥7 mm. Ovulation was then triggered with a single intramuscular injection of hCG (6,000–10,000 IU).

###### 2.2.2.3.2 Artificial cycle protocol

On menstrual cycle days 2–3, a baseline transvaginal ultrasound was performed to measure endometrial thickness. Oral estradiol valerate was initiated at a dose of 4–5 mg three times daily. Endometrial thickness and the absence of dominant follicular development were monitored periodically by ultrasound, and the estrogen dose was adjusted if needed. If endometrial thickness remained <8 mm, the dose was increased to 8 mg/day for one week. Once estrogen had been administered for ≥12 days and endometrial thickness reached ≥8 mm, progesterone supplementation was added.

###### 2.2.2.3.3 GnRH agonist down-regulation + artificial cycle protocol

On menstrual cycle days 2–3, a long-acting GnRH agonist (3.75 mg) was administered intramuscularly. After 28–30 days, serum hormone levels and transvaginal ultrasound findings were re-evaluated. If down-regulation criteria were met, oral estradiol valerate was started, following the same dosing schedule as described for the artificial cycle protocol (Section 2.2.3.2). Endometrial thickness was monitored periodically during estrogen treatment.

### 2.3 Assessment of pregnancy outcomes

Biochemical pregnancy was determined by serum β-hCG measurement 14 days after embryo transfer. Patients with a positive test continued luteal phase support. Clinical pregnancy was confirmed by transvaginal ultrasound 28 days after transfer, defined as the visualization of a gestational sac and yolk sac. The following outcome measures were calculated: Clinical pregnancy rate = (number of ongoing pregnancies/total patients in the group) × 100%. Embryo implantation rate = (total number of implanted embryos/total embryos transferred in the group) × 100%. Biochemical pregnancy rate = (number of biochemical pregnancies/total patients in the group) × 100%. Early miscarriage rate = (number of early miscarriages/total patients in the group) × 100%.

### 2.4 Data collection

The following variables were collected: age; BMI; prior pregnancy history; duration of infertility; infertility factors and type; serum levels of luteinizing hormone (LH) and follicle-stimulating hormone (FSH) on menstrual day 3; progesterone (P) and estradiol (E2) levels on the day of hCG trigger; anti-Müllerian hormone (AMH) level on the day of oocyte retrieval; total number of oocytes retrieved; number of high-quality cleavage-stage embryos; number of embryos cryopreserved; number of degenerate embryos; endometrial preparation protocol; total number of embryos transferred; type of embryos transferred; whether the transferred embryos were high quality; endometrial thickness (mm); endometrial pattern; number of endometrial blood vessels; endometrial blood flow grade; and Doppler parameters of the left and right uterine arteries, including S/D, RI, and PI.

### 2.5 Statistical analysis

Data were analyzed using SPSS 26.0. Continuous variables with normal distribution are presented as mean ± standard deviation and compared using one-way ANOVA; non-normally distributed continuous variables are reported as median (interquartile range) and analyzed with the Kruskal–Wallis H test. In post-hoc pairwise comparisons following ANOVA or the Kruskal–Wallis test, the Bonferroni correction was applied, with *P* < 0.008 considered statistically significant. Categorical variables are expressed as frequency (percentage) and compared using the chi-square test or Fisher’s exact test (when expected cell counts were <5), with *P* < 0.05 considered statistically significant. Forward stepwise logistic regression was performed to calculate odds ratios (OR) and corresponding 95% confidence intervals (CI).

## 3 Result

### 3.1 Comparison of baseline clinical characteristics among PCOS patients stratified by BMI

As presented in **Table 1**, statistically significant differences were observed across BMI groups in terms of infertility duration, E2 levels, and the number of high-quality embryos.

**Table 1:** Basic clinical characteristics of PCOS patients classified by BMI groups.

Variables	Total (n = 129)	Slim (n = 15)	Normal (n = 73)	Overweight (n = 30)	Obese (n = 11)	P
Age (y)	31.00 (29.00, 34.50)	30.00 (29.50, 31.50)	31.00 (29.00, 34.00)	31.50 (29.00, 33.00)	33.00 (31.00, 35.50)	0.311
Duration of Infertility(y)	3 (2, 4)	2 (1, 2)^cd^	3 (2, 4)	3 (2, 4)	6 (4, 8)^b^	<.001
Etiology of Infertility						0.082
Female Factor	61	4	41	11	5	
Male Factor	16	1	9	4	2	
Combined Factor	41	10	17	12	2	
Unexplained	11	0	6	3	2	
Infertility kind, n						0.149
Primary	77	9	38	21	9	
Secondary	52	6	35	9	2	
bAMH, ng/mL	7.48 (5.78, 10.71)	7.20 (5.98, 7.48)	7.09 (5.73, 9.26)	6.65 (5.27, 10.30)	5.20 (4.59, 12.02)	0.902
bFSH, mIU/mL	5.13 (3.65, 6.44)	5.09 (3.75, 7.39)	5.28 (3.90, 6.45)	4.77 (3.82, 6.39)	3.60 (2.60, 5.05)	0.124
bLH, mIU/mL	6.70 (4.87, 9.04)	8.32 (6.41, 10.76)	6.90 (4.87, 9.02)	6.11 (3.96, 8.32)	5.70 (5.10, 8.60)	0.137
LH/FSH	1.32 (1.03, 1.83)	1.75 (1.14, 2.27)	1.31 (1.05, 1.71)	1.27 (0.78, 1.79)	1.72 (1.41, 2.09)	0.127
bProg, ng/mL	0.28 (0.14, 0.84)	0.39 (0.21, 1.06)	0.33 (0.15, 0.79)	0.19 (0.13, 0.35)	0.28 (0.13, 0.68)	0.251
bE2, pg/mL	27.97 (23.92, 36.03)	40.10 (35.67, 48.28)^*^	28.01 (24.34, 34.06)	25.48 (22.68, 29.32)	23.05 (20.89, 25.18)	<.001
Endometrial preparation regimen, n						0.227
HRT	52	6	27	14	5	
GnRHa+HRT	25	3	13	4	5	
Modified natural cycle	52	6	33	12	1	
Number of Oocytes Retrieved	15 (11, 19)	15 (13, 16)	15 (12, 18)	16 (11, 20)	12 (9, 15)	0.247
No. of High-quality embryos	5 (4, 7)	4 (3, 6)	6 (4, 7)	5 (4, 7)	5 (5, 5)	0.036
Number of Cryopreserved Embryos	2 (1, 2)	2 (1, 2)	2 (1, 3)	2 (1, 2)	2 (2, 2)	0.121
Number of Degenerated Embryos	1 (0, 2)	1 (0, 2)	2 (0, 3)	1 (0, 2)	1 (0, 1)	0.058
No. of embryos transferred	1 (1, 2)	1 (1, 2)	1 (1, 2)	1 (1, 2)	1 (1, 2)	0.786
Embryo kind, n						0.892
Cleavage embryo	50	6	29	12	3	
Blastocyst	79	9	44	18	8	
Quality of the Transferred Embryos						0.068
High-Quality Embryo	90	11	55	20	4	
Transferable Embryo	39	4	18	10	7	

Different superscript letters (a, b, c, d) denote significant pairwise differences relative to the underweight, normal-weight, overweight, and obese groups, respectively. An asterisk (*) indicates a significant difference from all other groups.

For variables that showed overall significance in the Kruskal–Wallis H test, post-hoc pairwise comparisons were performed using the Dunn–Bonferroni method. The results revealed the following: Infertility duration was significantly shorter in the underweight group compared with the overweight group (adjusted *P* = 0.002) and the obese group (adjusted *P* < 0.001). The obese group had a significantly longer duration of infertility than the normal-weight group (adjusted *P* < 0.001). E2 levels were significantly higher in the underweight group than in each of the other three groups (adjusted *P* < 0.001).

### 3.2 Comparison of ultrasound parameters and pregnancy outcomes among PCOS patients stratified by BMI

Significant differences were observed in endometrial thickness across BMI groups (*P* = 0.011). Endometrial thickness in the normal-weight group was significantly greater than in the other three groups, while the obese group exhibited the thinnest endometrium among all groups. Endometrial morphology distribution also differed significantly among the groups (*P* = 0.034). The proportion of Type A endometrium was higher in the normal-weight group and lower in the overweight and obese groups. The number of endometrial blood vessels showed significant between-group variation (*P* = 0.014), with the fewest vessels observed in the obese group. The distribution of subendometrial blood flow grades differed significantly across BMI categories (*P* = 0.001). Post-hoc pairwise comparisons with Bonferroni correction revealed that the proportion of high-grade blood flow was significantly higher in the normal-weight group than in the obese group (adjusted *P* = 0.001). Uterine artery hemodynamic parameters also varied significantly among BMI groups. The S/D of uterine artery blood flow was significantly higher in the overweight group than in the normal-weight group (adjusted *P* = 0.007). Similarly, the PI of uterine artery flow was significantly higher in the overweight group compared with both the normal-weight and underweight groups (adjusted *P* = 0.001 for both comparisons). Notably, abnormalities in right-sided uterine artery parameters (PI, RI, and S/D) were generally more pronounced than those on the left side.

#### 3.2.1 Ongoing pregnancy rates across BMI groups

The ongoing pregnancy rates were as follows: 67.12% in the normal-weight group, 46.67% in the overweight group, 45.45% in the obese group, and 40.00% in the underweight group. The chi-square test indicated no statistically significant difference between groups (*P* = 0.081), though the normal-weight group demonstrated a trend toward higher ongoing pregnancy rates.

#### 3.2.2 Embryo implantation and early miscarriage rates

The embryo implantation rate did not differ significantly across BMI groups (*P* = 0.124); however, the highest implantation rate (66.67%) was observed in the normal-weight group. Similarly, the early miscarriage rate showed no statistically significant difference among groups (*P* = 0.575). Clinically, the obese group presented the highest early miscarriage rate (27.27%), which was approximately 2.2-fold higher than that of the normal-weight group (12.33%), suggesting a noteworthy trend despite the lack of statistical significance, as presented in **Table 2**.

**Table 2:** Ultrasound parameters and pregnancy outcomes of PCOS patients stratified by BMI.

Variables	Total (n = 129)	Slim (n = 15)	Normal (n = 73)	Overweight (n = 30)	Obese (n = 11)	P
Pregnancy outcomes						
Sustained pregnancy rate, n(%)	74(57.36%)	6 (40.00%)	49 (67.12%)	14 (46.67%)	5 (45.45%)	0.081
Embryo implantation rate, %	59.20% (103/174)	50.00% (10/20)	66.67% (66/99)	45.00% (18/40)	60.00% (9/15)	0.124
Biochemical pregnancy rate, n(%)	6 (4.65%)	1 (6.67%)	1 (1.37%)	3 (10.00%)	1 (9.09%)	0.228
Early miscarriage rate, n(%)	20 (15.50%)	3 (20.00%)	9 (12.33%)	5 (16.67%)	3 (27.27%)	0.575
Endometrial parameters						
Intimal thickness (mm)	8.40 (7.50, 9.40)	7.40 (7.00, 8.30)	8.80 (7.90, 9.50)	8.50 (7.70, 10.20)	7.10 (6.70, 8.05)	0.011
Intimal morphology, n (%)						0.034
A	21	3	11	6	1	
B	49	10	27	6	6	
C	59	2	35	18	4	
Endometrial blood flow quantity	4 (4, 6)	5 (4, 6)	5 (4, 6)	4 (3, 5)	3 (3, 4)	0.014
Endometrial blood flow grading						0.001
1	48	5	19	15	9	
2	49	8	27	12	2	
3	32	2	27	3	0^b	
Uterine artery blood flow parameters						
Left S/D	5.52 ± 1.60	4.97 ± 1.10	5.26 ± 1.44	6.26 ± 1.88	5.95 ± 1.76	0.010
Right S/D	5.71 ± 1.74	5.19 ± 0.79	5.47 ± 1.71	6.48 ± 1.89^b^	5.95 ± 1.87	0.028
S/D	5.61 ± 1.57	5.08 ± 0.83	5.36 ± 1.51	6.37 ± 1.73^b^	5.95 ± 1.72	0.010
Left RI	0.80 ± 0.05	0.78 ± 0.05	0.80 ± 0.05	0.83 ± 0.05	0.81 ± 0.06	0.017
Right RI	0.81 ± 0.05	0.79 ± 0.04	0.80 ± 0.05	0.84 ± 0.04	0.82 ± 0.06	0.013
RI	0.81 ± 0.05	0.79 ± 0.04	0.80 ± 0.05	0.83 ± 0.04	0.82 ± 0.06	0.006
Left PI	2.02 ± 0.44	1.84 ± 0.31	1.94 ± 0.44	2.23 ± 0.40^b^	2.22 ± 0.46	0.003
Right PI	2.08 ± 0.44	1.92 ± 0.23	2.00 ± 0.45	2.34 ± 0.40^ab^	2.15 ± 0.47	0.001
PI	2.05 ± 0.42	1.88 ± 0.25	1.97 ± 0.43	2.29 ± 0.36^ab^	2.18 ± 0.45	0.001

Different superscript letters (a, b, c, d) denote significant pairwise differences relative to the underweight, normal-weight, overweight, and obese groups, respectively.

### 3.3 Factors associated with embryo implantation in PCOS patients undergoing assisted reproduction

**Figure 2:**
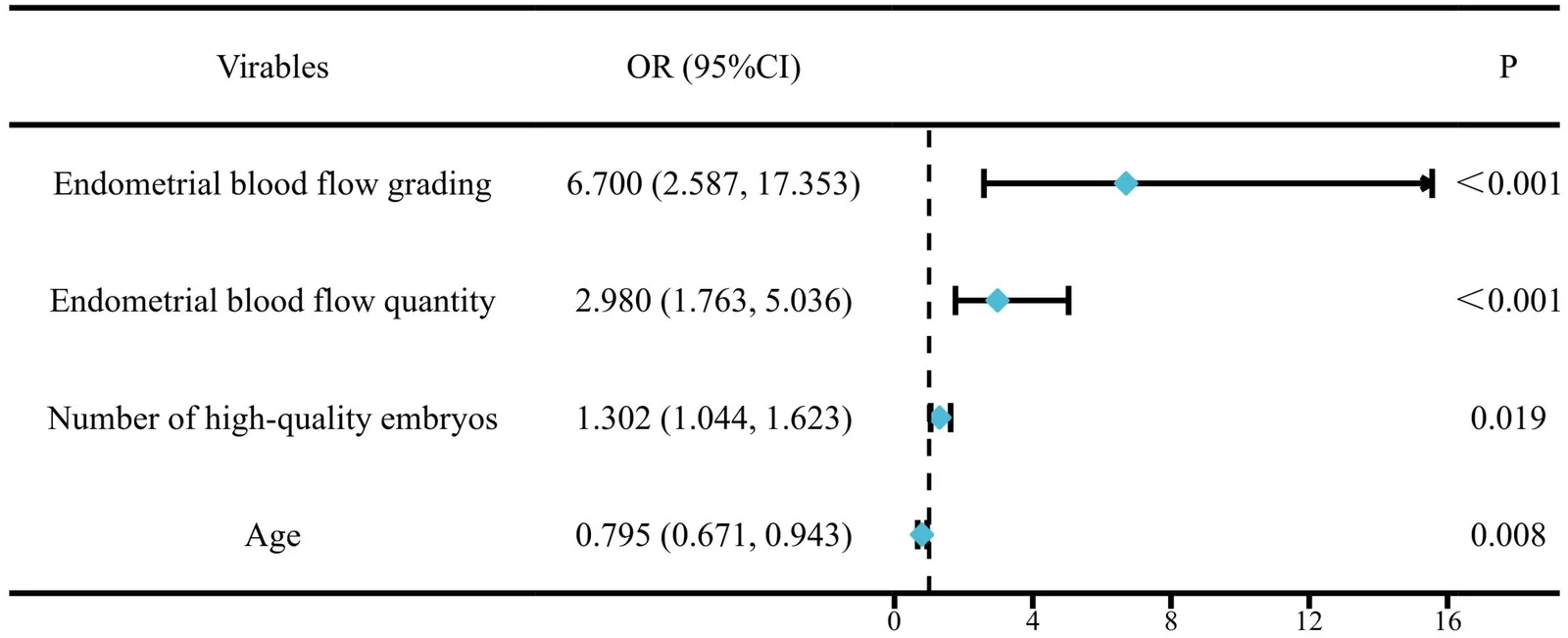
Logistic regression forest plot of factors associated with embryo implantation rate in patients with PCOS undergoing IVF-ET.

Logistic regression analysis identified several factors significantly associated with embryo implantation in PCOS patients following IVF-ET. Higher endometrial blood flow grade, greater number of endometrial blood vessels, and a higher number of high-quality embryos were independent protective factors for successful implantation (all *P* < 0.05). In contrast, advanced maternal age was a significant risk factor for implantation failure (*P* < 0.05). Among the significant protective factors, endometrial blood flow grade had the strongest association (OR = 6.700), followed by the number of endometrial blood vessels (OR = 2.980), as detailed in **Figure 2**.

The overall model demonstrated a good fit: the Hosmer-Lemeshow test (χ² = 8.691, *P* = 0.369) indicated no significant deviation from the observed data. The Nagelkerke R² value of 0.605 suggests that the model explains a substantial proportion of the variance in embryo implantation outcomes.

### 3.4 Predictive performance of ultrasound and clinical indicators for embryo implantation in pcos patients undergoing assisted reproduction

**Figure 3:**
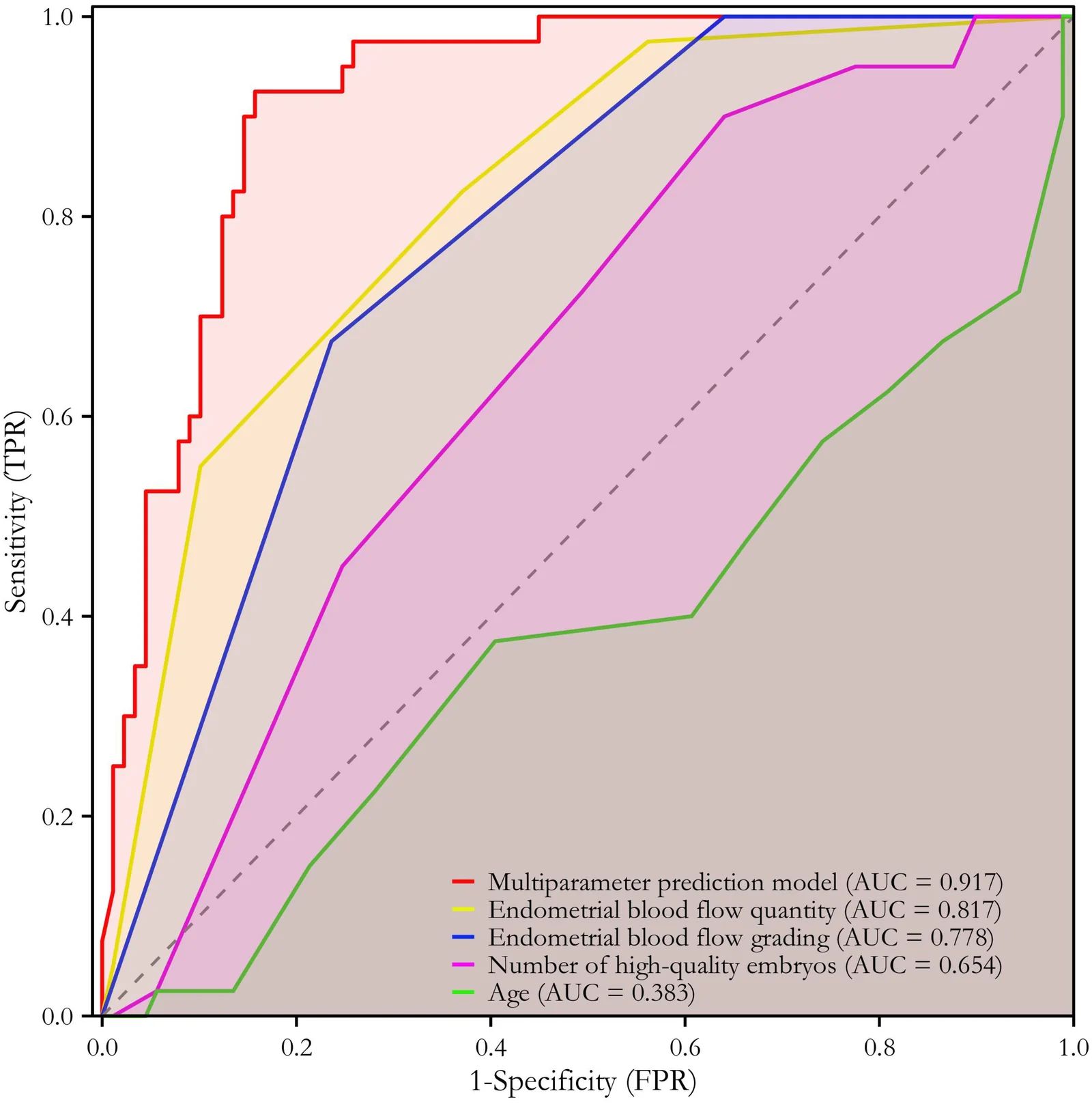
Predictive performance of individual and combined indicators for embryo implantation in patients with PCOS.

Using embryo implantation success as the outcome variable, receiver operating characteristic (ROC) curves were plotted to evaluate the predictive performance of endometrial blood flow grade, endometrial blood flow quantity, number of high-quality embryos, and patient age. The analysis revealed significant differences in the predictive ability of these variables. Endometrial blood flow grade demonstrated the highest predictive value, with an area under the curve (AUC) of 0.817 (95% CI: 0.742–0.893, *P* < 0.001). This was closely followed by endometrial blood flow quantity (AUC = 0.778, 95% CI: 0.699–0.857, *P* < 0.001). Among embryo-related parameters, the number of high-quality embryos showed moderate predictive efficacy (AUC = 0.654, 95% CI: 0.557–0.750, *P* = 0.005). In contrast, age exhibited the lowest predictive performance (AUC = 0.383, 95% CI: 0.273–0.493, *P* = 0.034). Notably, a multivariable predictive model incorporating the above key factors through logistic regression displayed substantially improved discriminative ability, achieving an AUC of 0.917 (95% CI: 0.870–0.964, *P* < 0.001). The predictive performance of this combined model was significantly superior to that of any single variable, underscoring its enhanced clinical utility for outcome prediction, as illustrated in **Figure 3**.

## 4 Discussion

By analyzing endometrial and uterine artery blood flow parameters in PCOS patients grouped according to BMI, this study demonstrates the influence of body weight on endometrial receptivity and uterine artery hemodynamics, offering important theoretical support for clinical practice. These findings underscore the value of preconception weight management in patients undergoing assisted reproductive technology.

The impact of obesity on endometrial receptivity is a complex, multifactorial issue, and current evidence remains somewhat controversial. On one hand, obesity is recognized as an independent risk factor for adverse pregnancy outcomes (9), which may impair endometrial function through multiple pathways and consequently reduce embryo implantation and pregnancy rates (10). On the other hand, some studies suggest that elevated BMI alone may not directly compromise endometrial receptivity (11). Therefore, this study focuses specifically on PCOS patients across a range of BMIs to investigate the effect of BMI on endometrial receptivity and uterine artery blood flow during ART cycles. The results are expected to provide a scientific basis for optimizing individualized treatment strategies in this patient population.

Previous studies have reported that approximately 38% to 88% of patients with PCOS are overweight or obese (12). In the present cohort, however, the proportion of obese PCOS patients was lower, at approximately 31.78%. This discrepancy may reflect distinct regional characteristics among women in southwestern China. First, women in this region tend to have a generally smaller body size, which may be influenced by genetic background (13). Second, regional factors such as climate, dietary habits (e.g., lower fat and higher fiber intake), and lifestyle (e.g., greater physical activity levels) may also contribute to the lower prevalence of obesity. Thus, although the obesity rate in this study is below some previously reported estimates, it remains plausible within the context of southwestern China. Future research could further examine how genetic, environmental, and lifestyle factors interact to influence obesity rates in PCOS patients.

The results of this study showed that the duration of infertility in the obese group was significantly longer than that in the normal-weight group (adjusted P < 0.001), suggesting that obese patients may delay seeking medical care due to excessive focus on weight loss. Previous studies have also reported that obese women often underestimate the impact of body weight on fertility and thus fail to pursue timely medical intervention (9). Of note, our study also observed an association between underweight status and elevated estradiol (E2) levels (14); however, the underlying mechanism remains unclear and requires further investigation.

Regarding ovarian function, although the between-group comparison of oocyte yield did not reach statistical significance in our study (P = 0.247), the median number of oocytes retrieved was lowest in obese patients (12 oocytes), representing a clinically meaningful trend. This finding is consistent with previous literature: studies have clearly reported that overweight and obese women have significantly fewer oocytes retrieved than normal-weight women (15, 16). More importantly, the number of high-quality embryos differed significantly across BMI groups, with the normal-weight group obtaining more high-quality embryos, suggesting that abnormal body weight may impair oocyte and embryo quality. Recent evidence from Iavarone et al. further provides complementary insights from the ovarian perspective: obese women exhibited not only significantly lower AMH levels but also a marked reduction in the ratio of mature oocytes to total FSH dose (MII/FSH ratio), confirming that obesity impairs oocyte maturation efficiency (17).

Combining these ovarian findings with our previous observations—that obesity reduces endometrial blood flow, increases uterine artery resistance, and decreases the proportion of Type A endometrium—offers a more comprehensive mechanistic picture: obesity compromises both the “seed” (oocyte/embryo quality) and the “soil” (endometrial receptivity), synergistically lowering embryo implantation rates. Therefore, overweight or obese PCOS patients should undergo comprehensive weight management before ART cycles to improve both ovarian response and the endometrial environment. Clinicians should strengthen health education for obese patients, emphasizing the value of early intervention (18) to shorten the duration of infertility and improve pregnancy success.

Although the ongoing pregnancy rate, embryo implantation rate, and early miscarriage rate did not differ statistically across BMI groups in this study (*P* > 0.05), the observed numerical trends merit further consideration. The normal weight group showed the most favorable outcomes across all indicators, a finding consistent with previous reports (16, 19, 20). The early miscarriage rate in the obese group was approximately 2.2-fold higher than that in the normal-weight group, aligning with the trend reported by Cui et al. (21), who identified obesity as a risk factor for miscarriage in PCOS patients. The significantly poorer endometrial blood flow and thinner endometrium observed in the obese group further suggest that obesity may contribute to early pregnancy loss.

The present study revealed significant differences in endometrial thickness, morphology, endometrial blood flow quantity and grading, and uterine artery hemodynamic parameters among PCOS patients of different BMI categories. Specifically, the obese group exhibited thinner endometrium, a lower proportion of Type A morphology, and reduced blood flow quantity, collectively indicating that obesity may impair endometrial receptivity through multiple pathways. These findings reflect the adverse influence of obesity on the endometrial microenvironment and suggest that clinical evaluation of obese women should include careful assessment of endometrial perfusion. Furthermore, the proportion of high-grade endometrial blood flow was significantly lower in the obese group than in the normal-weight group, further supporting the detrimental effect of obesity on endometrial physiology (22). Abnormal uterine artery hemodynamic parameters—notably elevated S/D and PI values—were particularly evident in overweight and obese groups, potentially linked to obesity-related endothelial dysfunction and chronic low-grade inflammation. Interestingly, abnormalities in right-sided uterine artery parameters (PI, RI, S/D) were generally more pronounced than those on the left side, which may reflect pelvic vascular anatomy or hemodynamic distribution patterns. This observation suggests that special attention should be paid to the right uterine artery during ultrasound evaluation. Notably, endometrial blood flow quantity in the underweight group did not differ significantly from that in the normal weight group, though endometrial thickness and morphology patterns warrant further study. This may indicate that both low and high BMI can adversely affect the endometrium, albeit through distinct mechanisms. The impact of underweight status on reproductive outcomes appears particularly complex. In summary, for obese patients with suboptimal endometrial perfusion, preconception interventions such as lifestyle modification (weight reduction), improvement of insulin sensitivity (e.g., with metformin), or medications aimed at enhancing endometrial blood flow (e.g., low-dose aspirin, vitamin E) could be considered prior to assisted reproductive treatment. These approaches may help inform the development of individualized management strategies.

The results of the logistic regression analysis indicate that higher endometrial blood flow grade, greater endometrial blood flow quantity, and a larger number of high-quality embryos are protective factors for successful embryo implantation following IVF-ET in patients with PCOS (all *P* < 0.05), whereas advanced maternal age is a risk factor for implantation failure (*P* < 0.05). These findings offer valuable evidence for further refining IVF-ET treatment strategies in this patient population. Further analysis revealed that endometrial blood flow grade had the strongest effect (OR = 6.700), followed by endometrial blood flow quantity (OR = 2.980), underscoring the central role of endometrial hemodynamics in the implantation process. Previous studies have established a positive correlation between adequate endometrial perfusion and embryo implantation rates (23, 24). The present study further confirms that favorable endometrial blood flow characteristics are significantly associated with IVF-ET success in PCOS patients, suggesting that clinical protocols should prioritize the evaluation and, where possible, enhancement of endometrial perfusion. Successful implantation after assisted reproductive technology largely depends on two key determinants: embryo quality and endometrial receptivity. Although the predictive value of the number of high-quality cleavage-stage embryos was modest in isolation, its contribution within the combined model remains relevant. Embryo quality continues to be a fundamental factor influencing pregnancy outcomes. Moreover, advancing age emerged as an independent risk factor for implantation failure, consistent with other reports (16, 25). While age alone showed limited predictive performance, it remains an important clinical indicator of reproductive potential, particularly in older patients. Consequently, for PCOS patients of advanced reproductive age, individualized treatment planning should be initiated as early as possible to optimize the likelihood of successful embryo implantation.

The predictive value of endometrial blood flow quantity, blood flow grade, and embryo-related parameters has been extensively studied in the context of assisted reproduction. This study further confirms that endometrial blood flow grade is the strongest individual predictor of pregnancy outcome, with an area under the curve (AUC) of 0.817, which is significantly higher than that of other single parameters. Notably, a multivariable prediction model integrating endometrial blood flow and embryo parameters achieved an AUC of 0.917, demonstrating significantly better discriminative ability than any single indicator. Based on the Youden index, the optimal cutoff value for the combined model was 0.768, yielding a sensitivity of 84.3% and a specificity of 92.5%. These results indicate that the model has high accuracy in distinguishing between successful and failed implantation. These findings underscore that pregnancy outcomes are not determined by a single factor but rather emerge from the interplay between embryo quality and the endometrial microenvironment. This supports the clinical utility of a multiparameter integrated assessment in ART practice. The proposed model can more accurately estimate the likelihood of embryo implantation, thereby offering a more reliable basis for clinical decision-making. Therefore, in clinical practice, combining endometrial blood flow assessment with embryo quality evaluation may allow more precise prediction of implantation success and help optimize IVF-ET outcomes for patients with PCOS. Future studies could explore the inclusion of additional potential predictors—such as endometrial receptivity biomarkers or embryo metabolomic profiles—to further refine the accuracy of prediction models.

This study offers several strengths: (1) it provides a systematic evaluation of the influence of BMI on endometrial thickness, morphology, blood flow distribution, and uterine artery hemodynamic parameters in PCOS patients; (2) it proposes an integrated predictive model for embryo implantation by combining ultrasound and clinical indicators. However, several limitations should be considered when interpreting the results. First, due to the single-center retrospective design and the fact that all participants were PCOS patients from southwestern China, the generalizability of our findings to PCOS populations of other ethnicities or geographic regions requires further validation. Second, although we adjusted for major confounders such as age, duration of infertility, and embryo quality, the retrospective design precludes us from completely excluding the potential impact of unmeasured confounding factors (e.g., detailed dietary patterns, psychological stress, and environmental endocrine disruptor exposure) on the results. Furthermore, certain potentially relevant variables—such as androgen levels, endometrial volume, and serum progesterone levels around the time of embryo transfer—were not available in a complete or standardized manner for all patients. In particular, although progesterone is recognized as a key modulator of endometrial receptivity, standardized data on peri-transfer progesterone levels (including timing of measurement, formulation, and route of administration for luteal phase support) were not uniformly available, precluding its inclusion in our analysis. Taken together, the conclusions of this study require further validation in large-scale, multicenter prospective studies that incorporate emerging assessment techniques. Future prospective studies should systematically collect the aforementioned unmeasured covariates, evaluate the interplay between BMI, progesterone levels, endometrial perfusion, and implantation outcomes, and include PCOS patients from diverse ethnic and geographic backgrounds, in order to provide more robust predictive models for clinical practice.

In summary, through analysis of ultrasound parameters—including endometrial thickness, morphology, and blood flow characteristics—as well as clinical outcomes across different BMI categories, this study underscores the importance of preconception weight management in PCOS patients undergoing ART. For individuals with abnormal BMI, fertility treatment cycles should include close monitoring of endometrial thickness, subendometrial blood flow, and uterine artery Doppler indices. Overweight patients merit particular attention to vascular resistance parameters, while obese patients may benefit from a more comprehensive approach aimed at optimizing endometrial receptivity. Weight management should be regarded as a core component of strategies to improve pregnancy outcomes in this population.

## Data Availability

The original contributions presented in the study are included in the article/supplementary material. Further inquiries can be directed to the corresponding author.
